# Increasing peak intensity of tropical cyclones passing through the Korean Peninsula

**DOI:** 10.1038/s41598-023-32020-w

**Published:** 2023-03-29

**Authors:** Joseph Basconcillo, Il-Ju Moon

**Affiliations:** 1 Department of Science and Technology, Philippine Atmospheric, Geophysical, and Astronomical Services Administration, Quezon City, Philippines; 2 Typhoon Research Center, Jeju National University, Jeju City, South Korea

**Keywords:** Climate change, Natural hazards

## Abstract

An increasing trend along with an abrupt increase in the peak intensity of tropical cyclones (TCs) passing through the Korean Peninsula (KP) are significantly detected from 1981 to 2020 and since 2003, respectively. Here we present observational evidence that such trend and shift are largely attributed with the increased passages of intense TCs in the KP during the mature boreal autumn (i.e., September–October, SO) and linked with the recent shift of the Pacific Decadal Oscillation (PDO) to its negative phase. A negative PDO during SO is related to environmental changes that are favorable for more intense TC passages in the KP including a weakened East Asian subtropical jet stream, weaker vertical wind shear, warmer subtropical sea surface temperature, and stronger low-level relative vorticity. Such findings are expected to provide new insights on understanding regional TC variability and ultimately, contribute to long range TC prediction initiatives in the KP region.

## Introduction

Tropical cyclones (TCs) are one of the most destructive natural hazards that pose serious threats to the lives and properties not only in the Korean Peninsula (KP) region but also in East Asia and in the other countries. Expectedly, an escalated TC-associated cost of damages is previously reported during the more active TC season (June to October, JJASO) and during the mature boreal autumn (September–October, SO), respectively, from 1981 to 2019 in East Asia^1^, which includes the KP region. Such increased TC-associated cost of damages have contributed to more than 39% of the total annual cost of damages associated with all hydrometeorological hazards in East Asia (i.e., flood, drought, heavy rainfall) in East Asia (hereafter, we refer to the said report as BM2022).

**Figure 1:**
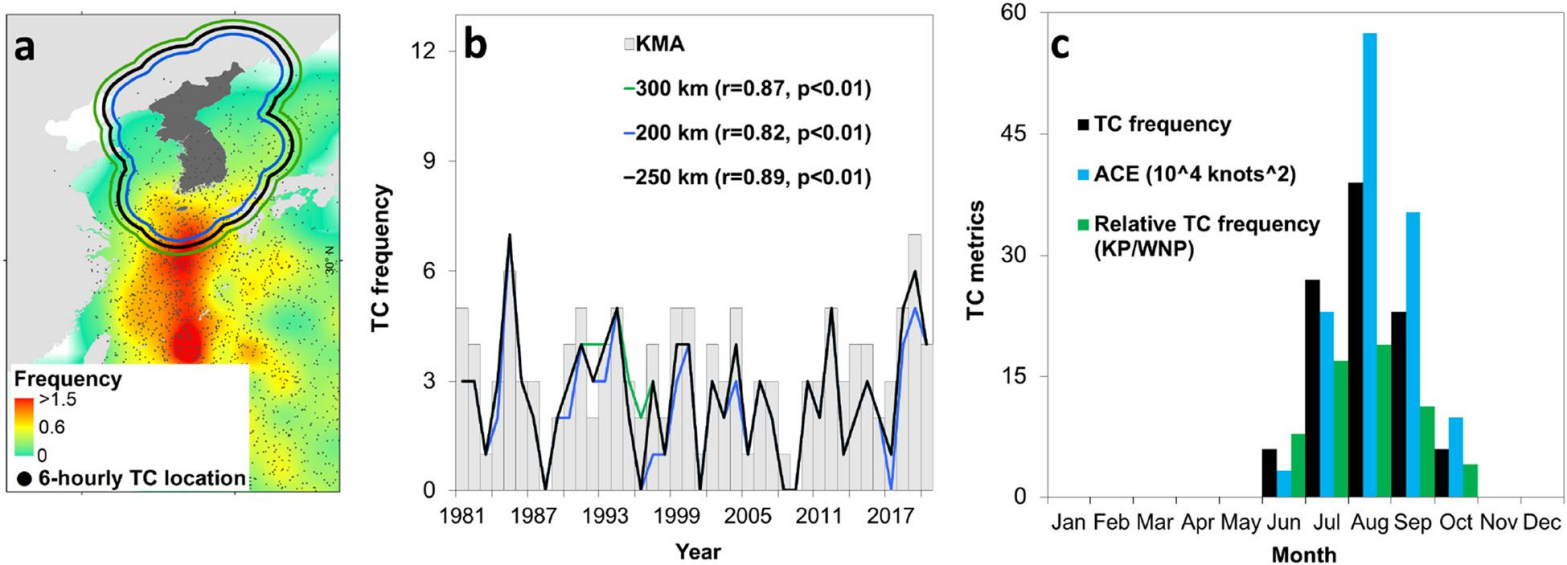
Climatology of tropical cyclones (TCs) passing through the Korean Peninsula (KP). (**a**) Climatological composite map of KP-influence TC frequency gridded into 2° × 2° horizontal resolution from 1981 to 2020. The green, blue, and black contour lines correspond to the 300, 200, and 250 km buffer zones away from the KP region (dark gray filled area), respectively. (**b**) Timeseries of frequency of KP-influence TCs based on the Korea Meteorological Agency (KMA, bar) and TC passage frequency in the 300 (green), 200 (blue), and 250 (black) km buffer zones, respectively. (**c**) Monthly mean of KP-influence TC frequency (black), Accumulated Cyclone Energy (ACE, blue), and relative KP-influence TC frequency with respect to the Western North Pacific (WNP) TC frequency (green), respectively. In (**a**) the map is plotted using ArcGIS 10.1 (https://www.esri.com).

Meanwhile, the TC season in the KP region runs in JJASO where its activity begins in June, peaks in August, and dips in October^2^. Here we refer to the TCs passing through the KP region as KP-influence TCs. For example, the TC tracks that passed through or intersect the delineated 250 km buffer zone away from the KP region are counted as KP-influence TCs (see black contour in Fig. 1a). As such, KP-influence TCs do not necessarily need to make a landfall anywhere in the mainland KP region to be counted in the timeseries of KP-influence TCs^2^ (Fig. 1b). Refer to the Methods for the detailed description of the KP-influence TCs and details of the other selected TC metrics used in our analysis.

Most KP-influence TCs occur during the boreal summer (i.e., June–August, JJA) and the rest are observed during the mature boreal autumn (i.e., SO)^3^ (Fig. 1c). There is no KP-influence TC observed during the months outside of the TC season such as May and November from 1981–2020, respectively. Similarly, the TC activity in the KP region, which can be heuristically represented by the Accumulated Cyclone Energy (ACE)^4^, also begins in June, peaks in August, and dips in October. About 19% or 27 out of 206 TCs in the Western North Pacific (WNP) enter the KP region in August—the highest monthly relative TC frequency (Fig. 1c). Meanwhile, the lowest monthly relative TC frequency values in the KP are observed in October at 4% (6 out 143 TCs) and 8% in June (6 out of 76 TCs), respectively. The relative KP-influence TC frequency during JJASO with respect to the WNP is 13% (101 out of 788 TCs) while the relative KP-influence TC frequency during JJASO with respect to the East Asian region is 23% (101 out of 435 TCs).

In terms of correlation, a previous study shows that there is low and insignificant correlation (R =  − 0.23) between KP-influence and WNP TCs from 1951 to 2008^2^. However, the reason and the underlying physical mechanism behind such low correlations were not addressed. Meanwhile, landfalling TCs in the KP from 1951 to 2004 are found to have increased since the late 1980s due to the changes in the Western North Pacific (WNP) subtropical high (WNPSH)^5–7^. Lastly, the heavy rainfall associated with landfalling TCs increased after the late 1970s^7^.

Previous studies further show that there is an increasing trend in TC landfall intensity in Korea and Japan (clustered together) that is linked to the strengthening of the WNPSH^5,8–11^. In addition, reports of increased TC destructive potential or Power Dissipation Index in East Asia attributed with the shifts of the Pacific Decadal Oscillation (PDO) to its negative phase have been presented^1,8–11^. It is described that a negative PDO is related to the weakening of the East Asian subtropical jet stream and the westward displacement of WNP subtropical high, which are found to be favorable for increasing TC passage frequency consequently leading to the increasing TC destructive potential in the East Asian region^1^. Some of the main intersections in these previous reports thought to influence the variability of KP-influence TCs include the displacement and variations of the WNPSH, the East Asian subtropical jet stream, and the subtropical north Pacific (e.g., PDO) and tropical sea surface temperature (SST) modes (e.g., El Niño Southern Oscillation)^1,8–11^.

While there are already several research on TCs done in the WNP and in the East Asian region^1,5–11^, there has been few studies that underscore the recent changes in the climatological characteristics of KP-influence TCs, particularly their intensity, which highlights the knowledge gaps on this subject. Because of their small number, most studies done in the KP region tend to spatially cluster KP-influence TCs with the entire WNP or East Asian region^1,8,10^. Temporally, the KP-influence TC season is often merged and considered as one season given the same reason—the low number of KP-influence TCs (only 19% of TCs in the WNP pass through KP in JJASO).

It is previously presented that there is a strong intraseasonality within the TC season in East Asia^1^. It is demonstrated that the Power Dissipation Index or TC destructive potential in JJA is not correlated and has opposing long-term trends and shifts with that in SO. This likely suggests that the TCs during the boreal summer (e.g., JJA) are not necessarily related with the TCs during the mature boreal autumn (e.g., SO), and vice versa^1^, which is likely due to the distinct large-scale environment between JJA and SO.

In addition, there is also prominent intrabasin TC variability in the WNP, which means that the mechanisms modulating TC variability in the entire WNP basin do not automatically apply in the smaller subregions of the WNP basin itself^1,12–18^. Previous studies have shown that the favorable environment for TC development is generally confined in the southeast quadrant of the WNP, which can be attributed with its covariability with known climate modes such as the El Niño Southern Oscillation, monsoon environment, and the Pacific Meridional Mode. For example, the genesis potential index during June-November, which heuristically quantifies the influence of large-scale environment on TC development, is found to be significantly correlated in southeast and northeast quadrants of the WNP but it has practically no correlation in the southwest and northwest quadrants of the WNP^12^. Such mechanism can be attributed with the spatial distribution of the warm/cold tongue of SST anomalies associated with the El Niño Southern Oscillation typically reach only the southeast quadrant of the WNP, thus, it has the strongest signal of interannual variability when compared with the other WNP quadrant^13^. During the strong WNP summer monsoon years, the monsoon trough extends eastward around 160°E^14^. In contrast, the monsoon trough retreats westward around 115° E during weak monsoon years. An eastward-extended monsoon trough provides a more moist environment and larger background for cyclonic systems, which are favorable for increased TC development^14–16^^.^ Meanwhile, the Pacific Meridional Mode, which is another tropical Pacific SST mode, is significantly correlated with the TC frequency in the southeast quadrant but not in the southwest quadrant of the WNP^17^. Such divergence in the intraseasonality and clustering of TCs lead to the dilution or simplification of understanding large-scale features which might not be applicable to the smaller subregions of the WNP such as the KP region, and the smaller time scale, respectively.

The recent passages of intense TCs in the KP region such as Super Typhoon Kong-Rey (2018), Super Typhoon Lingling (2019), and Super Typhoon Maysak (2020) bring into attention whether such intense TC passages are reflection of changes in the characteristics of KP-influence TCs. As of writing this manuscript, Super Typhoon Hinnamnor (2022) entered and made a landfall in September 2022 causing major impacts and damages to infrastructures and properties in the KP region^19^.

Emanating from such knowledge gaps, we investigate the climatological characteristics of KP-influence TCs especially in light of the recent passages of intense storms in the KP and the reported increasing damages associated with TCs^1^. This is because the KP region is one of the most robust national economies in the world ranking 13th globally and 4th in the Asian region in 2021^20^. This implies that, by extension, the KP-influence TC-associated cost of damages could pose considerable economic risks and losses not only in the KP region, but also globally, which consequently adds another layer of significance to our study. Through spatiotemporal and statistical analyses, we present observational evidence of the changes in the characteristics of KP-influence TCs with emphasis on their recent shifts and trends. Ultimately, we aim to provide new insights on the climatology of KP-influence TCs to support existing disaster risk reduction platforms in the KP region.

## Results

### Changes in the characteristics of KP-influence TCs

**Figure 2:**
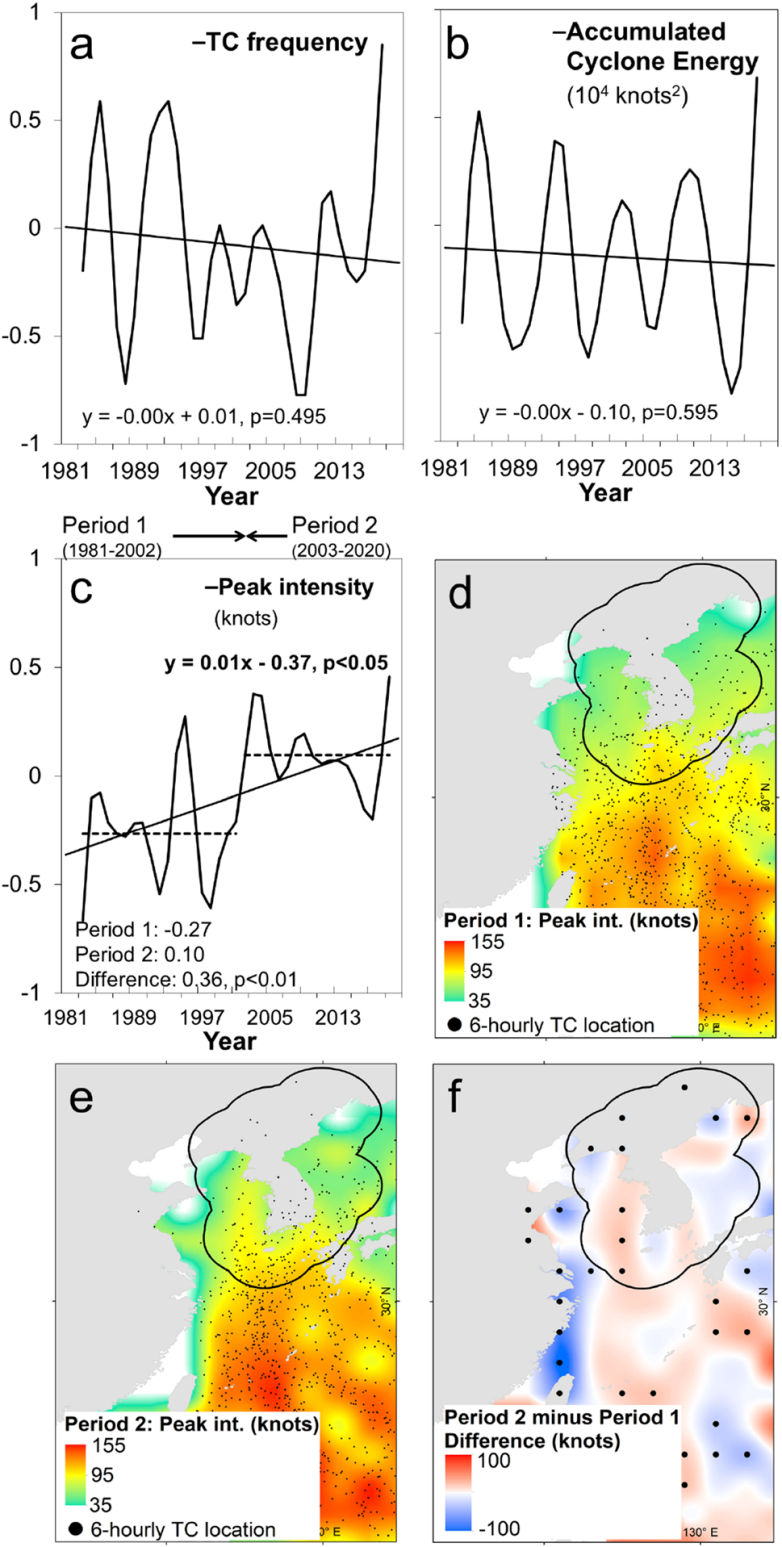
Increase in peak intensity of tropical cyclones (TCs) passing through the Korean Peninsula (KP). (**a**)–(**c**), Timeseries of indicated TC metrics during June-October. All timeseries are filtered using the 1-3-4–3-1 technique. In c, the dashed lines and inset statistics indicate the mean values of peak TC intensity during Period 1 (1981–2002) and Period 2 (2003–2020), respectively. (**d**), (**e**), Composite map of peak TC intensity gridded into 2° × 2° horizontal resolution during Period 1 (1981–2002) and Period 2 (2003–2020), respectively. The black dots indicate six-hourly TC location. (**f**) Composite difference in peak TC intensity between Period 2 and Period 1, respectively. The dots indicate significance at *p* < 0.05. In (**d**)–(**f**), the black contour line indicates the 250 km buffer zone away from the KP. In d-f, the maps are plotted using ArcGIS 10.1 (https://www.esri.com).

We limit our analysis to the JJASO season from 1981 to 2020 given that the KP-influence TCs only occur during this season^3^. Results show that there is no significant trend in the frequency of KP-influence TCs during JJASO (Fig. 2a). Meanwhile, the ACE is a widely-used heuristic metric of TC activity because it can account for TC duration, intensity, and frequency^4^. Results also show that there is no significant trend and abrupt regime shift in the ACE of KP-influence TCs during JJASO since 1981 (Fig. 2b).

When used in a composite timeseries (i.e., interannual, seasonal) the maximum intensity of TCs typically gets diluted because of the propensity to employ measures of central tendencies (i.e., mean, median, mode). Hence, we picked the maximum intensity of all TCs in a given season and/or year and referred to it as the peak TC intensity. For example, if a particular TC recorded a lifetime maximum intensity (LMI) of 100 knots and such value is the highest during that year and/or season then it is considered as the annual or seasonal peak TC intensity or the intensity of the strongest TC in the same year and/or season. Interestingly, results show that there is a significant trend in the peak TC intensity in the KP region from 1981 to 2020 (*p* < 0.05) during JJASO (Fig. 2c). Additionally, an abrupt increase in peak TC intensity is detected between Period 1 (1981–2002, x̄ =  − 0.27) and Period 2 (2003–2020, x̄ = 0.10, *p* < 0.01) during JJASO, respectively. Such significant trend and shift point to an increasing trend and abrupt increase in the intensity of strongest TCs crossing the KP region. For this reason, we used the peak TC intensity and its detected regime shifts between Period 2 and 1 in our succeeding analysis to characterize the changes in KP-influence TCs.

The spatial distribution of the peak intensity of KP-influence TCs during Period 1 is most intense in the southern portion of the KP region (Fig. 2d). Meanwhile, the spatial distribution of the peak TC intensity during Period 2 became more intense and prominent in the western seaboard and northern portions of the KP region, respectively (Fig. 2e) leading to the significant increase in the peak TC intensity in the said areas (Fig. 2f).

### Intraseasonality of peak TC intensity

BM2022 used the term intraseasonality to denote the pronounced difference in the characteristics of TCs within the entire TC season (i.e., JJASO) in East Asia. It is reported that the sources of TC variability in JJA are not necessarily applicable in SO even though both JJA and SO are found within the entire TC season, hence there is a within-season TC variability or intraseasonality. Following the approach in BM2022, we also divided the peak intensity of KP-influence TCs between JJA and SO, respectively. To the best of our knowledge, such an approach has not been done in TC studies in the KP region.

**Figure 3:**
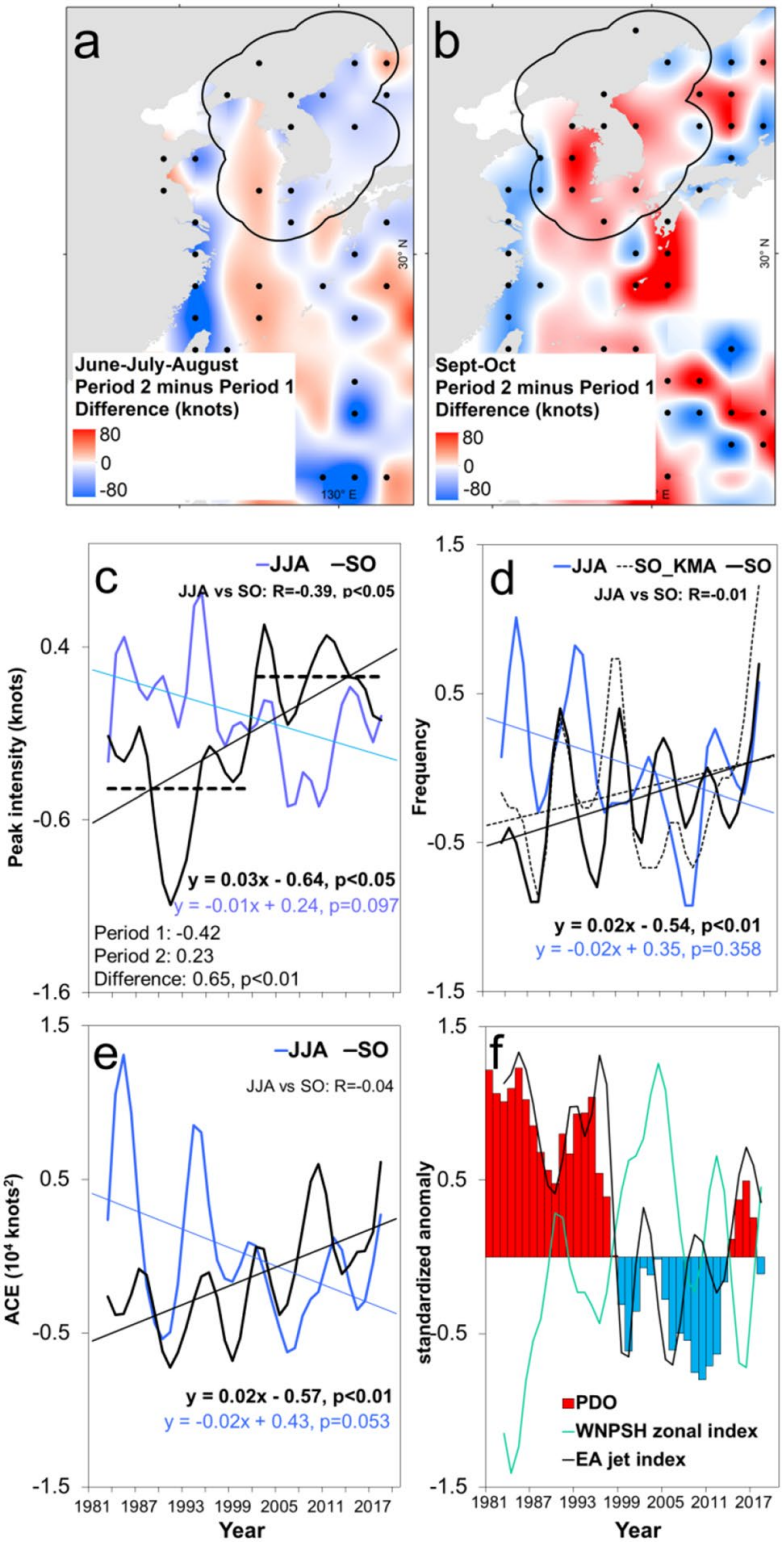
Trend and variability of tropical cyclone (TC) characteristics in the Korean Peninsula (KP). (**a**), (**b**) Composite difference map in the peak TC intensity gridded into 2° × 2° horizontal resolution between Period 1 (1981–2002) and Period 2 (2003–2020) in June–August (JJA) and September–October (SO), respectively. The dots indicate significance at *p* < 0.05. The black contour line indicates the 250 km buffer zone away from the KP. (**c**) Timeseries of peak TC intensity in JJA (blue) and SO (black), respectively. The dashed lines indicate the mean values of peak TC intensity during Period 1 and Period 2, respectively. The inset statistics show the mean values of peak TC intensity during Period 1 (1981–2002) and Period 2 (2003–2020), and their difference, respectively. (**d**), (**e**) Timeseries of TC frequency and Accumulated Cyclone Energy (ACE) in June–August (blue) and September–October (black), respectively. In d, the dashed line indicates the timeseries of TC frequency during SO from 1981 to 2020 based on KMA data. (**f**) Timeseries of the Pacific Decadal Oscillation (PDO, bar), East Asian subtropical jet stream index (black), and the Western North Pacific subtropical high zonal index (green), respectively. In (**a**), (**b**), the maps are plotted using ArcGIS 10.1 (https://www.esri.com).

The composite difference in the peak TC intensity between Period 2 and Period 1 is more apparent during SO when compared to JJA (Fig. 3a,b). The spatial distribution of the significant changes in peak TC intensity in SO becomes more widespread in the entire KP region and more prominent in its western seaboard unlike in JJA where the increases in peak TC intensity are generally confined only along a north to south-oriented swath in the western section of the KP region. Interestingly, the timeseries of the peak TC intensity in JJA and SO are also divergent and anticorrelated with each other (r =  − 0.39, *p* < 0.05) (Fig. 3c). Of the two seasons, only the peak TC intensity in SO has a significant trend (*p* < 0.05) along with an abrupt regime shift (*p* < 0.01) between Period 1 (x̄ =  − 0.42) and Period 2 (2003–2020, x̄ = 0.23), respectively (Table 1).

**Table 1:** Shift, trend, and correlation of indicated TC metrics and selected climate indices during the mature boreal autumn, respectively.

	Trend and shift					
	Peak TC intensity	TC frequency	Accumulated cyclone energy (ACE)	East Asian subtropical jet stream (EAJ) Index	Western North Pacific subtropical High (WNPSH) zonal Index	Pacific decadal oscillation (PDO)
Period 1 (Period 2)	− 0.42 (0.23)	− 0.33 (− 0.09)	− 0.37 (0.09)	0.69 (0.03)	− 0.25 (0.28)	0.58 (− 0.24)
Difference (*p*-value)	0.65 **(< 0.01)**	0.23 **(< 0.01)**	0.46 **(< 0.01 <)**	− 0.66 **(< 0.01)**	0.54 **(< 0.01)**	− 0.82 **(< 0.01)**
Trend (*p*-value)	0.03 **(0.021)**	0.02 **(< 0.01)**	0.02 **(< 0.01)**	− 0.03 (0.059)	0.03 (0.140)	− 0.05 **(< 0.05)**

	Bivariate correlation coefficients					
	TC frequency	ACE	EAJ index	WNPSH zonal Index	PDO	
Peak TC intensity (*p*-value)	− 0.11 (0.522)	0.75 **(< 0.01)**	− 0.38 **(< 0.05)**	0.23 (0.169)	− 0.57 **(< 0.01)**	
TC frequency (*p*-value)		− 0.07 (0.669)	− 0.52 **(< 0.05)**	0.52 **(< 0.01)**	− 0.42 **(< 0.05)**	
ACE (*p*-value)			− 0.10 (0.583)	0.04 (0.829)	− 0.44 **(< 0.01)**	
LMI (*p*-value)			− 0.21 (0.220)	0.31 (0.065)	− 0.13 (0.440)	
EAJ index (*p*-value)				− 0.78 **(< 0.01)**	0.86 **(< 0.01)**	
WNPSH zonal index (*p*-value)					− 0.68 **(< 0.01)**	

*p*-values in bold are significant at *p* < 0.05 confidence level.

We corroborate such findings by considering the frequency of the TCs and the mean intensity reaching or above the 90^th^ percentile of TC intensity climatology in JJA and SO, respectively (Supplementary Fig. 1a,b). Results also show the diverging timeseries in JJA and SO where significant shifts in the frequency of TCs (*p* < 0.01) and mean intensity *(p* < 0.01) of the upper 10% of intense TCs are detected during the Period 2, respectively. Therefore, such anticorrelation, trend, and shift support that the sources of TC variability during the SO are not necessarily applicable in JJA, thus, there is a prominent intraseasonality in the characteristics of KP-influence TCs between the said seasons. With this reason, we used the SO as the seasonal scale in our succeeding analysis.

### Changes in the other TC metrics

After confirming evidence of intraseasonality in the characteristics of KP-influence TCs, we proceeded in decomposing the TC passage frequency and ACE between JJA and SO, respectively (Fig. 3d,e). Results show that both TC passage frequency and ACE also exhibit similar intraseasonality. To start with, the TC passage frequency (*p* < 0.01) in SO is significantly increasing (*p* < 0.01) and has abruptly increased during the Period 2 (x̄ = -0.09) compared with Period 1 (x̄ =  − 0.33, *p* < 0.01), respectively (Table 1). The TC frequency in SO based on KMA data^3^ has also exhibited a similar decreasing trend, which further supports our findings. Meanwhile, the ACE in SO is also significantly increasing (*p* < 0.05) and has abruptly shifted during Period 2 (x̄ =  − 0.09) compared with Period 1 (x̄ =  − 0.37, *p* < 0.01), respectively. Furthermore, the relative frequency of KP-influence TCs with respect to the TCs in the WNP has similar diverging long-term trends during JJA and SO, respectively (Supplementary Fig. 2a,b). More particularly, the relative TC frequency in JJA is decreasing in contrast with the significantly increasing trend in SO (p < 0.01), which suggests that even though the total TC frequency is decreasing in the WNP, more TCs are passing through the KP region, especially during the SO. Therefore, the changes detected in the characteristics of KP-influence TCs including their activity, intensity, and passage frequency are mostly observed during the SO.

### Influence of the large scale environment

**Figure 4:**
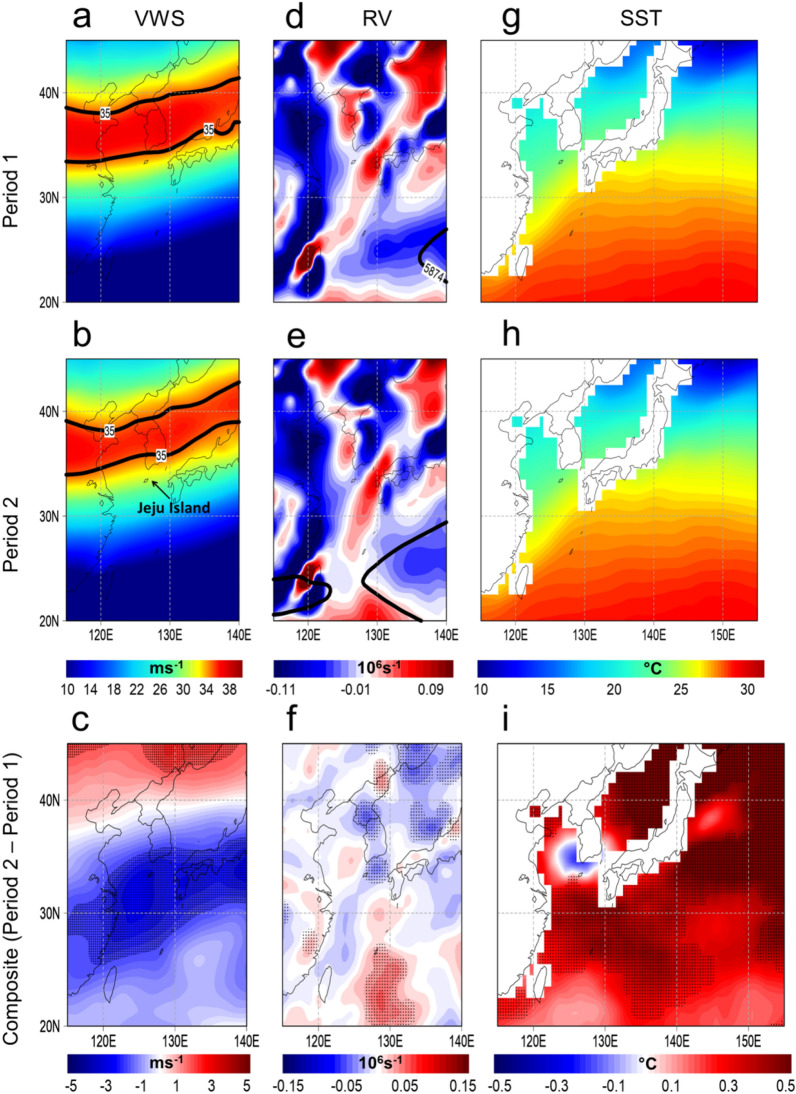
Changes in the large-scale environment. (**a**), (**d**), (**g**) Composite map of vertical wind shear (VWS), relative vorticity (RV), and sea surface temperature (SST) during the Period 1 (1981–2002) in September–October (SO), respectively. (**b**), (**e**), (**h**) same as (**a**), (**d**), (**g**) but for Period 2 (2003–2020) in SO, respectively. (**c**), (**f**), (**i**) Composite difference map in the indicated large-scale environmental parameters between Period 2 and Period 1 in SO, respectively. The dots indicate significance at *p* < 0.05. In a and b, black lines represent the contour line of VWS (35 ms^-1^) during Period 1 and Period 2, respectively. In d and f, black lines represent the location of the WNP subtropical high (= 5874 gpm) during Period 1 and Period 2, respectively. The maps are plotted using GrADS v2.2.1 (http://opengrads.org/).

To determine the influence of the large-scale environment, we plotted the composite difference maps in the 200–850 hPa vertical wind shear, 850 hPa relative vorticity, and SST between Period 2 and Period 1 in SO, respectively (Fig. 4a–i). Similar composite maps were made while removing the days of KP-influence TC occurrences to confirm that the detected changes in the large-scale environment are not caused by the TC passages themselves but rather highlight the robust changes in the background environment (Supplementary Fig. 3a–i). During Period 2, the 200–850 hPa vertical wind shear has weakened while the region of strong vertical wind shear has northward-shifted around 33°N in the southern portion of the KP region (Fig. 4a–c; for location reference of such changes, see Jeju Island in Fig. 4b when compared with Fig. 4a). A strong vertical wind shear induces asymmetry in the inner TC structure and restricts ventilation at the upper level that consequently reduces TC intensification^21^. Thus, the detected weakened vertical wind shear in the southern portion of the KP region during the Period 2 is a favorable environment for increased peak TC intensity in the KP region.

Note that the vertical wind shear is calculated using the difference between the upper level wind (200 hPa) and low-level wind (850 hPa). Because the vertical profile of wind speed decreases logarithmically with height, the upper level winds are generally stronger than the lower level winds. A decrease in the vertical wind shear results from a decrease in the upper level winds or an increase in the lower level winds, unless changes in wind direction are taken into account. In the KP region, a decrease in upper winds seems to lead to a decrease in the vertical wind shear.

**Figure 5:**
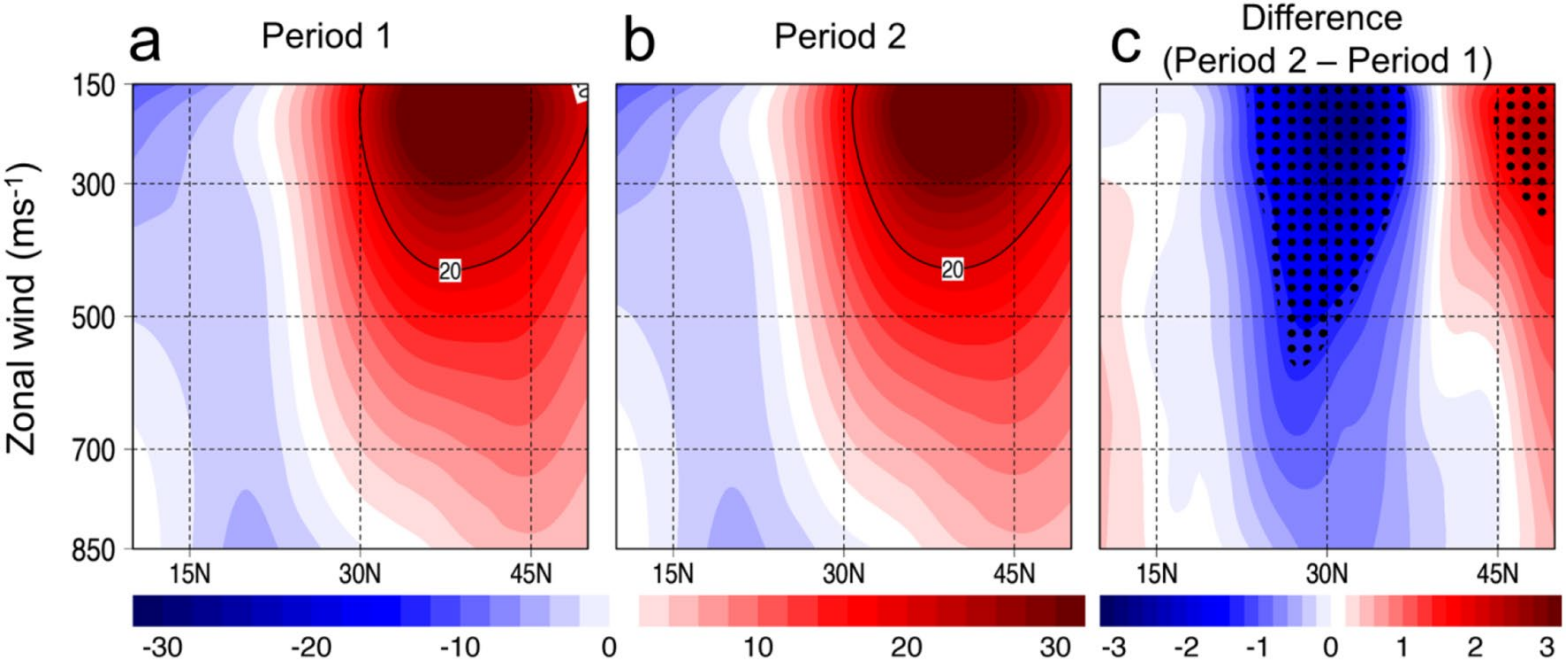
Changes in the East Asian subtropical jet stream. (**a**), (**b**) Composite vertical cross-section of zonal wind (850–150 hPa) averaged from 110° to 150°E during Period 1 (1981–2002) and Period 2 (2003–2020), respectively. (**c**) Composite difference between Period 2 and Period 1. The dots indicate significance at *p* < 0.05. The maps are plotted using GrADS v2.2.1 (http://opengrads.org/).

Consequently, the East Asian subtropical jet stream, which is typically prominent in the upper tropospheric level, has also significantly weakened during the Period 2 (Fig. 5a–c). It also appears that the East Asian subtropical jet stream not only has weakened but its southern flank has also shifted further north during the Period 2 approximately along 30°N between 100 and 300 hPa (see the contour line of 20 ms^-1^ in Fig. 5b). BM2022 has reported that the weakening of the East Asian subtropical jet during the SO is attributed with the recent shift of the PDO to its negative phase. Warmer SSTs in the subtropical north Pacific are conducive for convective activities, which may stall and weaken the East Asian subtropical jet stream. Meanwhile, a recent report on the weakening of the Eurasian subtropical jet (note that Eurasian region extends from central Asia to the western fringe of East Asia) shows that the reduction of aerosol concentration in the midlatitude resulting to more pronounced warming, which leads to a weaker meridional temperature gradient that consequently weakens the subtropical jet stream in the Eurasian region. Such reports possibly contribute to the recent weakening of the subtropical jetstream in the East Asian region. The strong horizontal upper level wind associated with the East Asian subtropical jet stream may inhibit TC passages and intensification, thus, a weakened and northward-shifted East Asian subtropical jet stream is favorable for the passages of more intense TCs in the KP region. This is further supported by the abrupt weakening of the East Asian subtropical jet stream^22^ index during Period 2 (x̄ = 0.69) compared to Period 1 (x̄ = 0.03, *p* < 0.01) (Table 1, see black line in Fig. 3f).

Meanwhile, the WNP subtropical high (WNPSH) has become westward-displaced during the Period 2 (Fig. 4d,e). The displacement of the WNPSH is denoted by the positive 850 hPa relative vorticity anomalies (or anomalous 850 hPa cyclonic vorticity) in the vicinity of the Okinawa Islands (Fig. 4f), which are favorable background environments for TC passages heading north towards the KP region. The WNPSH zonal index has also abruptly increased during Period 2 (x̄ =  − 0.25, *p* < 0.01) (Table 1, see green line in Fig. 3f) compared to Period 1 (x̄ = 0.28). A positive WNPSH zonal index means more westward location of the WNPSH^23^, which steers TC passages in the KP region and corroborates the detected westward-displacement of the WNPSH during Period 2. Previous reports show evidence of recent decadal changes in the WNPSH, particularly its westward displacement. The ocean warming over the tropical Indo-Pacific since the late 1970s led to more convection in the same area and weakened the westerlies, which become more favorable for the westward extension of the WNPSH^24,25^. Additionally, the observed increase in precipitation over the East Asian summer monsoon region led to increased evaporation and moisture flux in the East Asian region, which enhanced the ridge of the WNPSH in the 2000s towards the northwestward direction^26^. Note that such described changes in the large-scale environment are similarly observed in Supplementary Fig. 3a–i.

The westward displacement of the WNPSH is generally not conducive for TC development, which explains the decreasing TC frequency in the WNP during SO (Supplementary Fig. 2a). To corroborate, the correlation of the WNPSH zonal index and WNP TC frequency during SO is R =  − 0.53 (*p* < 0.01). An extended high pressure region, represented by the WNPSH, is characterized by prevalent dry conditions typical of an anticyclonic environment, which is not conducive for the TC development^1^. Furthermore, a previous study showed that the intensity of the WNPSH is significantly correlated with TC occurrences in East Asia, which means that as the WNPSH becomes intense, more TCs will pass through the East Asian region^27^. Such a relationship underscores that even though the WNP TC frequency is decreasing, it remains imperative to understand intrabasin and sub-regional TC variability (such as the KP-influence TCs) given the shifts in the large-scale features.

Of the two above mentioned indices, we note that the East Asian subtropical jet stream index is more correlated with the peak TC intensity (r = -0.38, *p* < 0.05) than the WNPSH zonal index (Table 1). However, the East Asian subtropical jet stream index is also well anticorrelated with the WNPSH zonal index (r =  − 0.78, *p* < 0.01), which means that although the East Asian subtropical jet stream index is more correlated with the peak TC intensity, we cannot ultimately discount the influence of the WNPSH zonal index considering that both indices remains well correlated with each other. Furthermore, we iterate that both indices have abruptly shifted during Period 2 (Fig. 3f, Table 1) indicating their respective influence in the detected shifts in the peak intensity of KP-influence TC during the Period 2.

### Linkage with long-term climate variability

BM2022 provided evidence that the increasing TC activity in East Asia is linked with the recent shift of the PDO to its negative phase during the SO^1^. The PDO is a leading long-term mode of variability of subtropical SSTs north of 20°N in the Pacific Ocean^28^. It is thought to have a high and low frequency periodicity of 15–25 years and 50–70 years, respectively^29,30^. The negative phase of PDO is primarily manifested through the warm SST anomalies in the subtropical North Pacific extending from the eastern Pacific to the East Asian region, which is related to the SST warming in the subtropical WNP between Periods 2 and Period 1 (Fig. 4g–i). Meanwhile, a positive PDO phase is characterized by reverse anomalous SST patterns.

We also show that the PDO during the SO is significantly anticorrelated with the peak TC intensity (r =  − 0.57, *p* < 0.01) as well as with the TC frequency (r =  − 0.42, *p* < 0.05) and ACE (r =  − 0.44, *p* < 0.01), respectively (Table 1). Such negative correlations indicate that the negative phase of the PDO results in the increase in peak intensity, passage frequency, and ACE of KP-influence TCs. While the PDO has already shifted to its negative phase in 1998 (Fig. 3f), we note that there was a short hiatus in the negative PDO phase until the early 2000s but it continued to be in the more prominent negative PDO phase thereafter. Such a hiatus could explain why the detected regime shifts in the peak intensity of KP-influence TCs started in the early 2000s (Figs. 2c, 3c, Supplementary Fig. 1b). However, it has to be emphasized that the PDO remains significantly different between Period 2 (x̄ = 0.58, *p* < 0.01) and Period 1 (x̄ =  − 0.24), which corroborates that our detected regime shifts are influenced by the PDO.

Moreover, it is reported that the influence of a negative PDO phase in TC characteristics in East Asia (including the KP region) is primarily manifested through the increased convection in the East Asian region acts to stall the East Asian subtropical jet stream, which favors the westward displacement and shifts in the WNPSH^24–26^ leading to the anomalous cyclonic vorticity in the vicinity of the Okinawa Islands and westward intrusion of the environmental steering flow^1^. Such a relationship is corroborated by the significant anticorrelation between PDO with the East Asian subtropical jet (r = 0.86, *p* < 0.01) and the WNPSH (r =  − 0.68, *p* < 0.01) in Table 1, respectively.

In summary, the warm SST anomalies in the North Pacific associated with a negative PDO phase lead to anomalous rising motion and formation of more convective activities in the same region^1,31^. Such rising motion and convective activities stall and weaken the East Asian subtropical jet stream allowing the westward displacement of the WNPSH, and westward intrusion of the environmental steering flow, which are all favorable and responsible for the passages of more intense TCs in the KP region. Such mechanisms explain why the negative PDO phase is anticorrelated and has modulated the recent changes in the characteristics of KP-influence TCs.

## Discussion

The growing impetus for more accurate information in long-range and operational TC forecasting allows us to revisit previous studies founded on larger spatial and temporal scales that are possibly less relevant in the demand of the present times—that is providing information for regional and smaller regions. For example, the limited available reports on KP-influence TCs are attributed to its small size and less TC passages in the KP relative to the entire WNP. Here we demonstrate that it remains imperative to characterize the climatology of KP-influence TCs, specifically, to understand the reasons and mechanisms that explain the recent passages of intense TCs in the region. We further argue that with the advent of more accessible information and big data, our approach can be replicated in the other subregions of the WNP such as the Philippine Sea and South China Sea, respectively, in the hope that we gain more insights on intraregional and intraseasonal TC characteristics that were previously overlooked before. As a matter of fact, BM2022 has also discussed that there is a decreasing TC destructive potential in Southeast Asia and in the Open Sea during the SO, which corroborates the need to diversify our understanding of intraseasonal and intraregional TC variability.

We also show that while the changes in the peak TC intensity are also found during the entire TC season (e.g. JJASO), most of the changes in investigated TC metrics such as peak intensity, frequency and ACE are significantly detected during the SO. Therefore, it becomes imperative to shift the focus of long-range and operational TC forecasting on the intraseasonality of KP-influence TCs. More particularly, we intend to pursue investigation of TCs during the boreal autumn globally in view of our findings.

We do not provide explicit attribution to anthropogenic climate change. A more immediate concern, however, lies on the shift of the PDO to its positive phase and whether such shift would result in different trends and shifts in the characteristics of KP-influence TCs. Moreover, our observational analysis does not explore the linkage of KP-influence TCs with the other climate known modes. We intend to leave such attribution and linkages in our future investigations.

Lastly, we expect that our findings on the recent shifts and trends in the characteristics of KP-influence TCs, most particularly in more intense TCs, can provide more insights in understanding TC variability and support disaster risk reduction not only in the KP region to WNP as well.

## Methods

### Tropical cyclone data

We used the TC best track data from the International Best Track Archive for Climate Stewardship (IBTRACS) version 4 from 1981 to 2020^32^. Only named TCs of tropical storm strength that first developed in the WNP with at least one six-hourly 35 knots maximum sustained wind during its lifetime were considered in our analysis. If a particular TC weakens below 35 knots during its passage in KP, we include it in our analysis of TC frequency as long as its track is still in the KP region.

### Scale of analysis and TC metrics

KMA maintains a monthly frequency of TCs that passed or influenced the KP region. Meanwhile, to identify the number of TCs that have influenced the KP region using the IBTrACS, we identified three geodesic buffer zones that are 200 km, 250 km, and 300 km away from the nearest coastlines of the KP (Fig. 1a), respectively. The said buffer zones are considered as proxy to the degree of influence of each TC away from the KP where increasing distance indicates diminishing influence. One count of TC means that the track of a particular TC during its lifetime intersected or passed through the said buffer zones. We counted and compared the total number of TCs that intersected or entered each buffer zone using the IBTRACS and the TC frequency data from KMA^3^ during JJASO (Fig. 1b). Results show that the TC passage frequency from the IBTRACS using the 250 km buffer zone has the highest correlation with the KMA data at r = 0.89 (*p* < 0.01). Such values are followed by the TC passage frequency using the 300 km (r = 0.87, *p* < 0.01) and 200 km (r = 0.82, *p* < 0.01) buffer zones, respectively. Therefore, our selected domain in TC analysis is the 250 km buffer zone. The TCs that intersected the 250 km buffer zone are referred to as KP-influence TCs.

Meanwhile, the countries’ boundary data used in our analysis are obtained from the DIVA-GIS platform and are not an explicit reflection of current or past geopolitical boundaries^33^.

We only selected KP-influence TCs during June-October (JJASO) season because the TC passages and TC activity in KP region only occur during this season (Fig. 1c). Specifically, the season of a TC is attributed to the time of its passage in the KP. For example, a TC that entered the KP in September is counted under the September–October season even though it developed in the WNP in August because our analysis covers TC passages in the KP region and not the genesis time itself. Unless specified, all TC timeseries and associated indices in our analysis were filtered using the 1-3-4-3-1 technique to minimize the influence of interannual variability^34^. This filtering technique uses a 5-value running centered approach where the middle value is multiplied with 4, the two neighboring values are multiplied with 3, and the distant neighboring values are taken as is. The product is divided by 12 to get the 1-3-4-3-1 filtered value.

The selected TC metrics used in our analysis are defined as follows:

- *TC passage frequency* count of TCs that passed through the 250 km buffer zone^32^
- *Peak TC intensity (knots)* highest six-hourly maximum sustained wind value of all TCs in a given season or year^32^
- *Accumulated Cyclone Energy (10*^4^* knots*^2^*)* sum of the squared of six-hourly maximum sustained wind greater than or equal to 35 knots of a TC over its lifetime^4,32^
- *Lifetime maximum intensity (knots)* highest six-hourly maximum sustained wind attained by a TC over its lifetime^32^

### Climate indices and reanalysis dataset

We used the Japanese Reanalysis 55-year (JRA-55) Project for atmospheric reanalysis and the Centennial in-situ Observation-Based Estimates SST for SST in our observational analysis^35^.

The vertical wind shear during SO is calculated using the mean wind speed difference at 200 hPa and 850 hPa. The East Asian subtropical jet stream index during SO is defined as the domain average of 300 hPa horizontal wind speed defined at 25°–40°N to 110°–150°E^22^. The WNPSH zonal index during SO is defined as the areal-averaged 500 hPa relative vorticity^23^ defined at 20–30°N, 120–135°E. The five-year running mean Pacific Decadal Oscillation (PDO) index during SO is obtained from the Physical Science Laboratory of the National Oceanic and Atmospheric Administration^36^.

### Statistical tests and grid visualization

The temporal bivariate correlation coefficients are measured using the Pearson’s correlation where their significance is tested in a two-tailed distribution. The significance of difference in the sampled composite means is tested using the student's t-test in a two-tailed distribution. The Rodionov algorithm is used to detect regime shifts^37,38^ in the indicated 1-3-4-3-1^18^ filtered timeseries with a cut-off length of 10 and significance at *p*<0.01 level. The detected regime shifts in the annual peak TC intensity are designated as Period 1 (1981-2002) and Period 2 (2003-2020), respectively.

Finally, the six-hourly location values of the indicated TC metrics were converted into 2° × 2° horizontal resolution grid and subsequently rasterized using bilinear interpolation technique for grid visualization.

## Supplementary Information

Supplementary Information.

## Data Availability

The reanalysis (https://data.ucar.edu/en/dataset/jra-55-japanese-55-year-reanalysis-monthly-means-and-variances) and best track (https://www.ncei.noaa.gov/products/international-best-track-archive) data products used in the analysis and described in the Methods are available for download from their indicated respective websites.
