# Supplementary material for: Increasing peak intensity of tropical cyclones passing through the Korean Peninsula
Source: Sci Rep. 2023 Mar 29;13:5097. doi: 10.1038/s41598-023-32020-w (PMC10060215; doi:10.1038/s41598-023-32020-w)
Supplement: Supplementary file 1 — Supplementary Information. [file 41598_2023_32020_MOESM1_ESM.docx]

**Increasing peak intensity of tropical cyclones passing through the Korean Peninsula**

Joseph Basconcillo^1, 2,^ Il-Ju Moon^2^

^1^Philippine Atmospheric, Geophysical, and Astronomical Services Administration, Department of Science and Technology, Quezon City, Philippines

^2^Typhoon Research Center, Jeju National University, Jeju City, South Korea

**Supplementary Information**

This Supplementary Information includes:

Supplementary Figure 1. Characteristics of tropical cyclones (TCs) passing through the Korean Peninsula with intensity above the 90^th^ percentile climatology

Supplementary Figure 2. Relative frequency of tropical cyclones (TCs) passing through the Korean Peninsula (KP) with respect to the Western North Pacific (WNP)

Supplementary Figure 3. Changes in the large-scale environment without tropical cyclone days

**
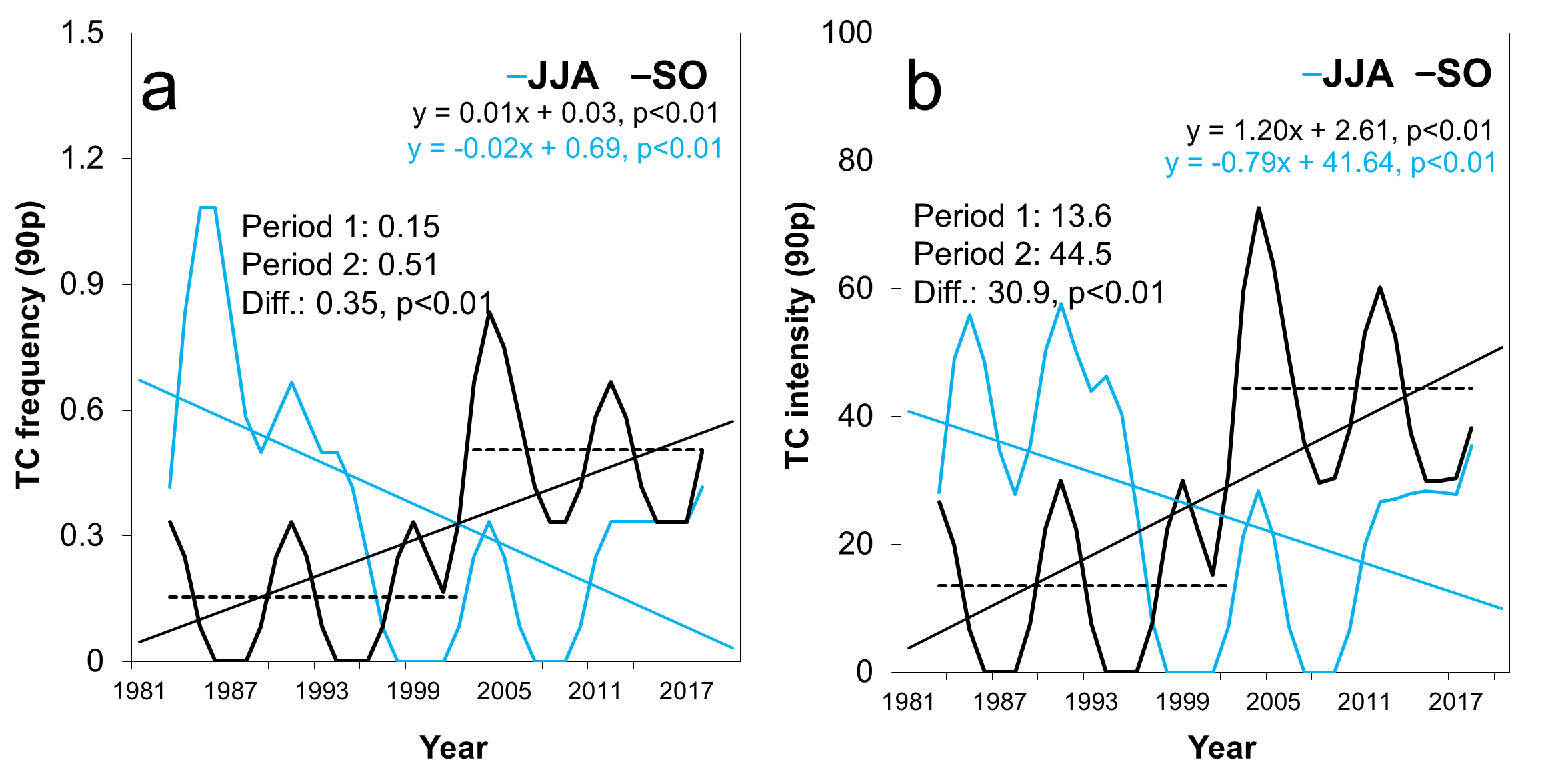
Supplementary Figure 1. Characteristics of tropical cyclones (TCs) passing through the Korean Peninsula with intensity above the 90^th^ percentile climatology. a,** Timeseries of KP-influence TC frequency reaching or above the 90^th^ percentile intensity from 1981-2020 during June-August (JJA, blue) and Sept-Oct (SO, black), respectively. The dashed lines indicate the mean values of KP-influence TC frequency during Period 1 (1981-2002) and Period 2 (2003-2020), respectively. **b,** as in **a** but for the mean TC intensity of KP-influence TCs reaching or above 90^th^ percentile intensity, respectively. In **a-b**, the inset statistics show the mean values of indicated TC metric during Period 1 (1981-2002) and Period 2 (2003-2020), and their difference, respectively.


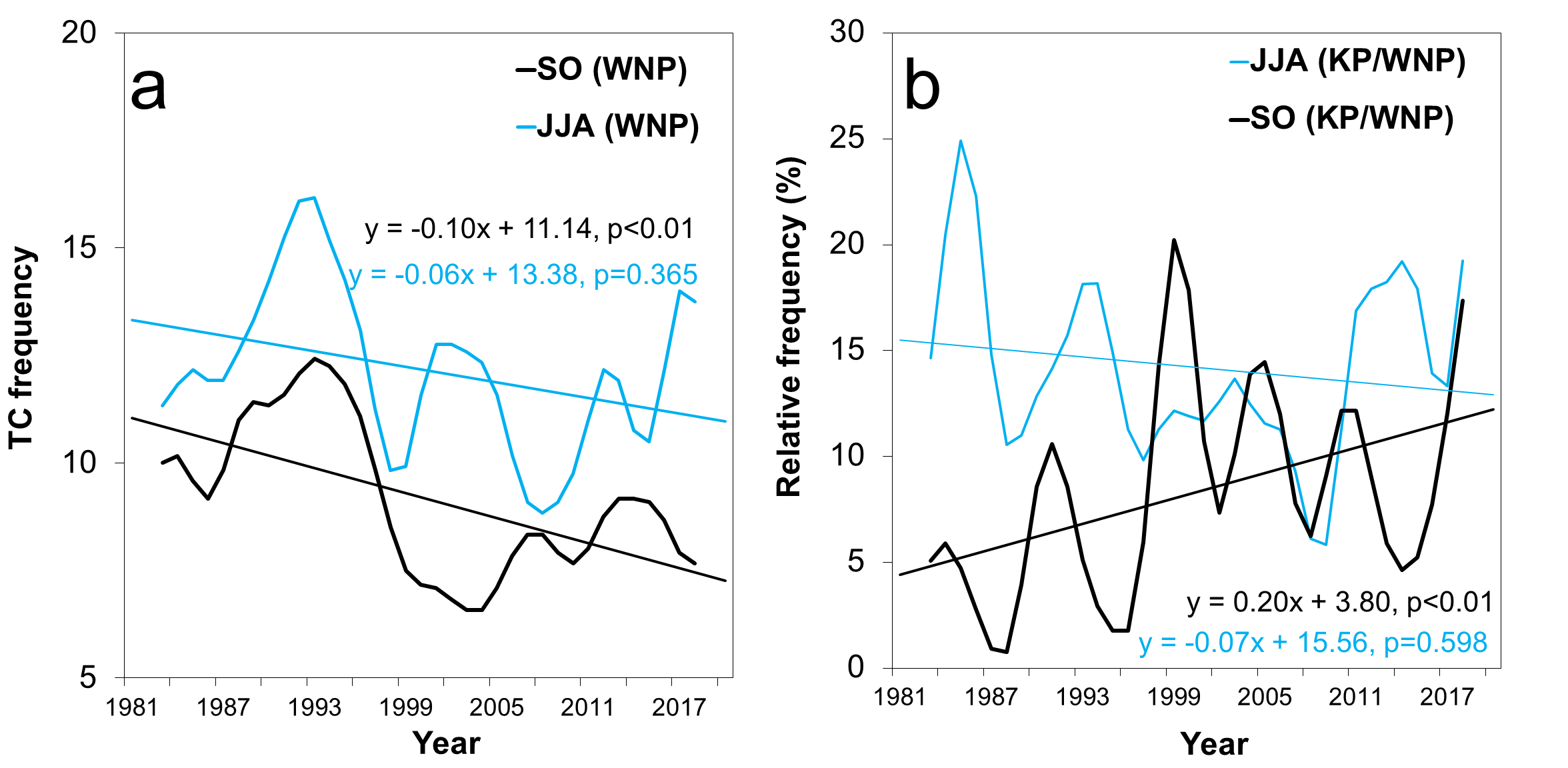
**Supplementary Figure 2. Relative frequency of tropical cyclones (TCs) passing through the Korean Peninsula (KP) with respect to the Western North Pacific (WNP). a,** Timeseries of TC frequency in the WNP from 1981-2020 during June-August (JJA, blue) and Sept-Oct (SO, black), respectively. **b,** Timeseries of relative TC frequency in the KP with respect to the WNP from 1981-2020 during June-August (JJA, blue) and Sept-Oct (SO, black), respectively


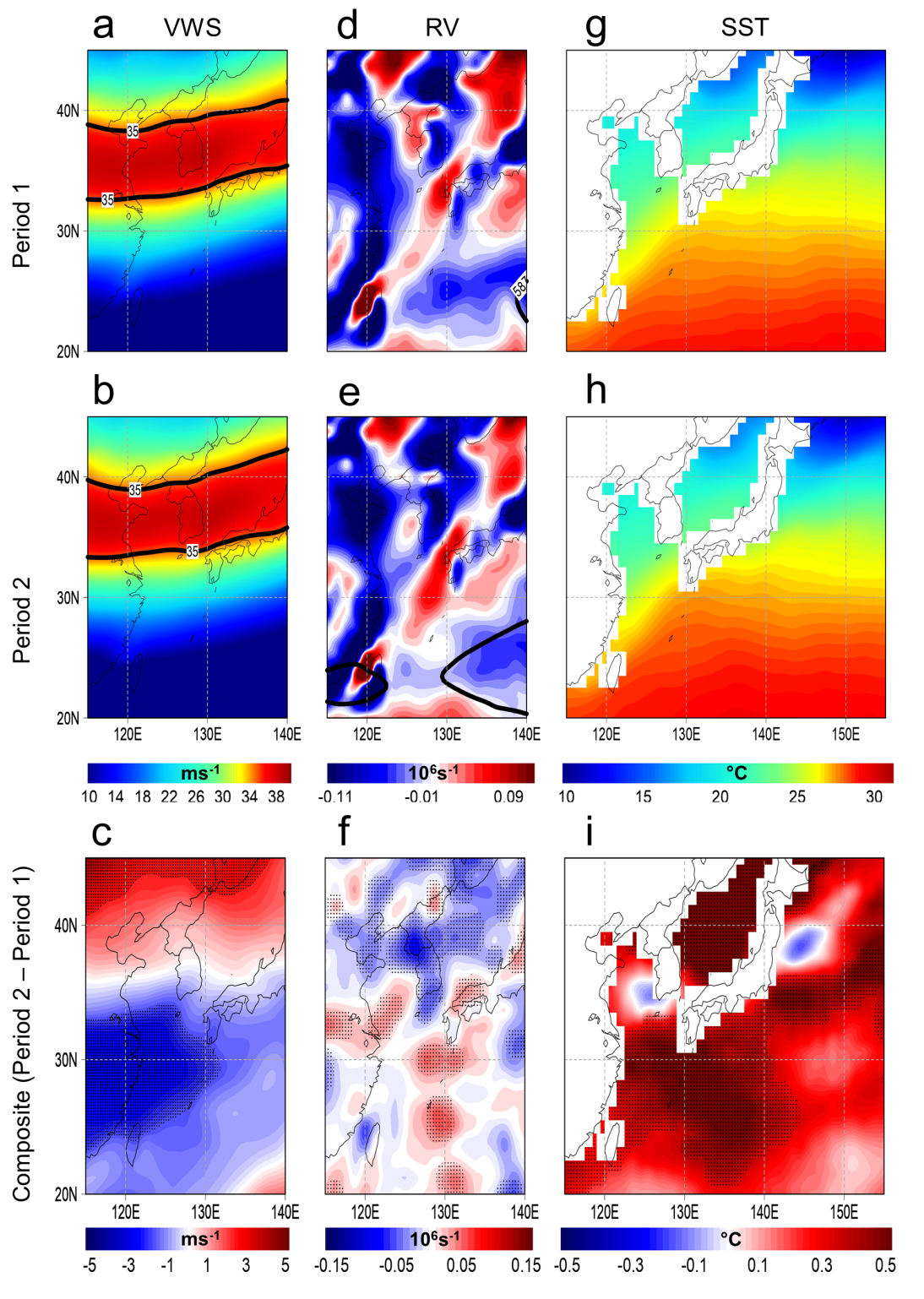


**Supplementary Figure 3. Changes in the large-scale environment without tropical cyclone days. a,d,g**, Composite map of vertical wind shear (VWS), relative vorticity (RV), and sea surface temperature (SST) during the Period 1 (1981-2002) in September-October (SO), respectively. **b,e,h**, same as **a,d,g** but for Period 2 (2003-2020) in SO, respectively. **c,f,i**, Composite difference map in the indicated large-scale environmental parameters between Period 2 and Period 1 in SO, respectively. The dots indicate significance at p<0.05. In **a** and **b**, black lines represent the contour line of VWS (35 ms^-1^) during Period 1 and Period 2, respectively. In **d** and **f**, black lines represent the location of the WNP subtropical high (= 5874 gpm) during Period 1 and Period 2, respectively. The maps are plotted using GrADS v2.2.1 (<http://opengrads.org/>).
